# Three ways of organising general practitioner’s medical services in sheltered housing. A qualitative study

**DOI:** 10.1080/02813432.2023.2256381

**Published:** 2023-09-14

**Authors:** Laila Tingvold, Line Melby

**Affiliations:** Centre for Care Research East, Norwegian University of Science and Technology (NTNU), Trondheim, Norway

**Keywords:** General practitioner (GP), sheltered housing, older patients, qualitative research

## Abstract

**Objective:**

Explore care providers’ experiences with the organisation of the medical services for residents in round-the-clock staffed sheltered housing.

**Design:**

Qualitative study and thematic analysis of individual interviews after strategic sampling of participants.

**Setting:**

Round-the-clock staffed sheltered housing in seven municipalities, inhabited by various user groups, and GPs in various locations in Norway.

**Subjects:**

In-depth interviews with 18 participants: 11 managers or employees in sheltered housing and seven GPs.

**Main outcome measures:**

Main themes and subthemes reporting participants’ experiences of medical provision to sheltered housing residents.

**Results:**

Three main models of organizing medical services for round-the-clock staffed sheltered housing were identified: (i) the ‘multiple GP’ model, where each resident has their own individual GP; (ii) the ‘single GP’ model, where all residents in the sheltered housing have one common GP; (iii) the ‘hybrid’ model, where a few dedicated GPs follow up the residents.

**Conclusion:**

Residents in round-the-clock staffed sheltered housing constitute a varied group that generally has substantial medical assistance needs. Given that many residents lack autonomy to manage their own care needs and make decisions, models with fewer GPs like models ii and iii seem to provide a better medical professional offer. Moving towards such an organising of the medical services for sheltered housing residents could have implications for GPs’ workload and competence needs. Future studies are needed to test models and assess implications.

## Abstract (plain language summary)

Residents in round-the-clock staffed sheltered housing are considered ‘home residents’ and consist of various user groups with extensive and often complex medical care needs.

The GP scheme is the most important medical service for home residents.

There is an emerging mismatch between the need for help of residents in sheltered housing and the GP scheme, and municipalities seek to remedy this by developing their own ad hoc solutions.

There is a need for a more systematic approach to deal with the medical needs of residents in round-the-clock staffed care homes.

## Introduction

The health- and care services are constantly evolving. Research and policy directives accompanied by financial incentives encourage municipalities to strengthen or develop some services, while others are left unchanged or reduced. In the Norwegian context, the development of sheltered housing is claimed to be probably the most significant change long-term care (LTC) services have undergone in recent times [1]. In the last 20 years, the number of sheltered housing residents has increased [2], in terms of both total number and relatively to residents in nursing homes [3]. During planning, sheltered housing was originally targeted for elderly people with somatic and cognitive challenges, constituting an extra step on the care ladder before nursing homes. Economic incentives to build sheltered housing instead of nursing homes motivated municipalities to also develop sheltered housing for other user groups than the elderly. In evaluating Care Plan 2015, managers in LTC services also considered sheltered housing a more dignified solution to care than nursing homes [4]. Sheltered housing is increasingly planned for users with psychiatric diagnoses or addiction problems and people with physical and/or intellectual disabilities. Nevertheless, elderly persons with diverse and complex healthcare needs constitute a large proportion of residents [5].

Sheltered housing residents are defined as ‘home dwellers’ and pay rent to the municipality which owns the housing. They also pay their own bills for medicines and receive home-care services according to their needs. Residents are legally considered ‘home dwellers’ and are therefore expected to make their own choice of a general practitioner (GP), like any other home-dwelling citizen. The GPs are responsible for following up residents’ medical needs. This stands in contrast to residents in nursing homes who are attended to by dedicated nursing home physicians and do not choose their physician.

Over recent years, differences between sheltered housing and nursing homes have become unclear, and responsibility is blurred [6,7]. Sheltered housing residents are increasingly sicker and in need of more advanced care [8] and treatment [4,9,10] which raises questions of the continuity of care [11]. This has led to concerns regarding whether sheltered housing residents receive the medical services they need and whether the current organization of healthcare services matches their needs and their ability to utilize the services [12].

### Sheltered housing

In Norway, sheltered housing emerged under the Action Plan for Elderly Care [13] and continued further into Care Plan 2015 [14], to provide more people with the opportunity to have a home adapted for comprehensive care. From 1994, grants were provided by the Housing Bank, and sheltered housing was defined as a ‘home that is adapted for people with disabilities and which is physically adapted so that the residents can receive round-the-clock care and attention’ [15]. At first, this involved ensuring universal accessibility for residents with impairments and that home-care staff could transport necessary equipment and assistive devices to the buildings.

Health- and care services are offered to residents based on individual needs. Initially, residents were served by the home-care service (Ministry of Health and Care Services, 2010–2011). With sheltered housing’s development, municipalities started to group together residents with similar care needs, finding it beneficial (practically and financially) to serve them simultaneously. Since 2010–2011, some municipalities have started hiring staff in some sheltered houses during daytime and afternoons or round the clock on a 24/7 basis. This was in response to some residents’ requirement for more care and support than home-care services could provide during daytime or on short visits. Since sheltered housing was also made available to residents with more and complex care needs, various staffing solutions have emerged. Some sheltered houses have hired permanent staff, with care services offered round the clock or daytime only. These adaptations are a result of the municipalities’ more practical approach to residents’ needs. From the evaluation of Care Plan 2015, it emerged, for example, that of municipalities that have received investment grants for sheltered housing, 38% have permanent full-time staff corresponding to those of nursing homes, 25% have permanent full-time staff daytime and availability *via* call center at nights, 23% have some sheltered housing with permanent full-time staff and some without permanent full-time staffing, while 10% responded that the care homes built with the investment subsidy do not have permanent full-time staffing [4]. It follows from the complexity of staffing that follow up of residents in sheltered housing requires collaboration across providers. Integrated care on the clinical level [16] might both affect patient experiences and cost-effectiveness, but good collaboration and care integration does not come by itself [17,18].

### GP schemes

GPs have medical responsibility for sheltered housing residents. The GP scheme was introduced in 2001 and gave everyone who lives in a Norwegian municipality the right to have a regular, named GP. The health authorities’ goal was to provide the population with a permanent physician over time, who knew the patient and their history. It was considered good medicine and good economics, with a fixed point of contact and continuity, simultaneously limiting ‘doctor shopping’ for sick leave and the prescribing of addictive drugs. The regular physician was also to communicate with therapists/other providers in hospitals and other health services, including by referring to the place of treatment and by sending discharge notes after treatment ended. Currently, the GP scheme in Norway is under pressure. The recent assessment shows that GPs experience a severe workload. They are obliged to perform more tasks than previously, and patients are sicker, thus requiring more advanced follow-up. Approximately 10% of GPs are considering quitting, and there are few medical students (approx. 9%) who want to work as GPs after finishing medical school. Put shortly, Norway may face a serious shortage of GPs. This will have consequences for the inhabitants, particularly those who are less able to request the services from a GP by themselves [19].

There is a limited amount of research conducted on residents in sheltered housing, and on care provision for this group. One strand of research focuses on the residents’ health status and needs. A Swedish study [20] found that residents in sheltered housing rate their health, quality of life and functional status lower than those aging in place, and that they have substantial care needs. The study concludes there is a lack of knowledge about what resources and services residents in sheltered housing use and need. A study from Ireland compared older people living in standard homes to residents in sheltered housing and found that living in sheltered housing complexes provided significant benefits for the well-being of residents if the housing had an on-site manager [21].

Nilsen et al. (2020) investigated factors affecting *pro re nata* (PRN) medication in sheltered housing [22]. PRN medication management was among other factors affected by health care personnels working relationships with other staff including GPs, as well as interactions with residents and their relatives, an issue which subsequently may affect residents’ outcomes. The authors found that the number of GPs related to the sheltered housing which were studied varied from 2 to 30, and the level of cooperation with health care personnel varied. Health care personnel saw themselves as being important spokespersons for the residents, particularly for those who rarely received medical follow-up by their GP [22]. Finally, studies have questioned if sheltered housing is suitable for frail elderly people in their last years of life [5] and if the living arrangements suits the aging population in rural areas with regards to safety and evolving care needs [7].

In the context of the development described above, with more sheltered housing being ‘the home’ for an increasingly frail and complex user group, combined with a GP scheme under strong pressure, we conducted a study to investigate the medical provision to sheltered housing residents.

The aim was to explore care providers’ experiences with the organisation of the medical services for residents in round-the-clock staffed sheltered housing.

## Methods and materials

The study had a qualitative, explorative design. A qualitative approach was chosen since it is best suited to embrace experiences and to explore fields where we have limited knowledge [23].

### Setting and sampling

The study setting was sheltered housing with round-the-clock staff in Norwegian municipalities. We applied a strategic approach when sampling municipalities and participants. We included municipalities of different sizes and locations in Norway and resident groups of various types. Furthermore, we strategically selected sheltered housing where we had reason to believe that medical services were organized in various ways [24–26]. Table 1 shows the included municipalities and the characteristics of the sheltered housing.

**Table 1.** Characteristics of the sheltered housing included in the study.

Municipality	Municipality size	Group of residents
1	30,000	Elderly, dementia, frail
2	20,000	Addiction and psychiatry
3	35,000	Residents with intellectual disabilities
4	3,500	Elderly, dementia, frail
5	40,000	Elderly
6	80,000	Elderly, light dementia
7	15,000	Disabled and multidisabled persons

The purpose of the qualitative research interview is to gain an understanding of various aspects of the participant’s situation and to present this from the person’s own perspective [27]. For recruiting participants, we contacted The Norwegian College for General Practice (Norsk forening for allmenmedisin) and two of the Development Centres for nursing homes and home care services. They suggested people who had knowledge about the study topic and who could have an interest in taking part in the study. They were contacted by the researchers and given information about the study. In total, 18 people agreed to participate. 11 sheltered housing managers or employees and seven GPs were interviewed.

Table 2 shows the study participants and their distribution over the seven included municipalities. Table 3 shows key characteristics for the GPs included in the study.

**Table 2.** Study participants, sheltered housing.

Municipality	Study participant	Professional background	Participant number
1	Manager sheltered housing	Nurse	1
	Ward manager sheltered housing	Nurse	2
2	Ward manager sheltered housing	Nurse	3
3	Manager unit sheltered housing unit	Social educator	4
	Manager unit sheltered housing unit	Nurse	5
4	Manager home-care services	Nurse	6
	Employee home-care / sheltered housing	Nurse	7
5	Municipal health manager	–	8
	Manager home-care services	Nurse	9
6	Manager sheltered housing	Nurse	10
7	Manager sheltered housing	Social scientist	11

**Table 3.** Study participants, general practitioners.

GP	Gender	Age	Years as GP	Number of patients on list	Participant number
1	F	60–70	5–10	Unknown	12
2	F	40–50	5–10	500	13
3	M	40–50	10–15	1400	14
4	M	40–50	10–15	1500	15
5	F	60–70	25–30	1200	16
6	M	60–70	30–35	Unknown	17
7	F	40–50	Unknown	N/A	18

We developed semi-structured interview guides for the target groups in the study. They were drafted and discussed between the researchers, before finishing them. The interview guides contained a combination of closed and open-ended questions, with greatest emphasis on open-ended questions. The advantage of open-ended questions is that the interviewer will receive a larger description of a situation, which in turn can lead to new knowledge [27]. Furthermore, the interviewer can ask follow-up questions based on the participant’s answers and explore issues that were not thought of initially.

The interviews, which were conducted between August 2020 and January 2021, lasted between 20 and 70 min. Both authors participated in data collection. All interviews were audiotaped and, except for one, transcribed verbatim.

### Analysis

The transcribed material includes about 190 pages in total. The analysis followed a bricolage approach [28], which is a common and eclectic mode of interview analysis. It implies the use of several techniques during the analysis, for example counting incidences and categorizing statements in pursuit of generating meaning of the complete material. In this study we leaned on concepts from the integrated care literature, such as continuity of care and collaboration, as a point of departure for the analysis. Both researchers read through the interview transcripts and discussed themes across the material. An overarching theme that was clearly present through the analysis was that there were pros and cons with various forms of organising. In addition, we identified cross cutting themes, including the value of continuity of care, and how collaboration between providers could affect resident follow up – themes that are familiar within integrated care. In addition, the individual’s right to choose GP emerged as a central theme during the analysis. We decided to focus on the pros and cons related to organising as the main theme because this seems to present a fundamental issue in terms of how to follow up the growing population of home-dwelling people in need of medical services.

### Ethics

The study was reported to the Norwegian Centre for Research Data (NSD), permit number 759739. All study participants agreed to be interviewed. Names and exact locations of the sheltered housing are anonymized.

## Results

Two clear-cut models of organizing the medical services were identified in the analysis. In addition, we found two cases of ad hoc organizing for responding to residents’ needs. These two are considered examples of what we may consider one model. Summarized, the analysis led to descriptions of the following three models for organizing the medical services for sheltered housing residents.

- Model 1 – the multiple GP model: Each resident has their individual GP
- Model 2 – the single GP model: One specific GP provides medical services for all residents
- Model 2 – the hybrid model: A few dedicated GPs provide medical services for residents

In the following, we present the three models, as described by the study participants. Common themes across all the models were the value of continuity of care, patients’ right to choose their own GP, and collaboration between GP and staff in the sheltered housing. These themes will be addressed below.

### Model 1. The multiple GP model: each resident has their individual GP

The first model is the traditional way of organizing medical services for sheltered housing residents. Here, each resident has their individual GP, in line with the fact that, in law, sheltered housing residents are home-dwelling persons and, consequently, entitled to choose their own GP. Several study participants argued that this model – especially if the GP has treated the resident for many years – ensures continuity of care, which is of great value when it comes to maintaining good health and function for the resident. Continuity provides the GP with accumulated patient knowledge over a long period of time and enables them to assess the resident’s state of health based on observations and health information gathered over several years. A GP claimed:

If the GP scheme works, then there is continuity in the follow-up. And we know that we have the greatest value for the patient. It can be shown in studies that continuity results in lower mortality… and this also applies when you come to an institution. So, knowing the patient’s medical history, knowing next of kin has great value… (Participant 18)

Another GP claimed that most of her patients preferred to continue contact with her as their GP when becoming sheltered housing residents. She said:

My impression is certainly that patients who live in sheltered housing still have a very clear wish to relate to me as their GP, who has, in a way, attended to them over several years. So, I think there is a certain wish among residents to still be allowed to choose. (Participant 13)

For residents with addiction and substance abuse problems, the freedom to choose their GP was claimed to be particularly important by several study participants. This group had often experienced traumas and usually had preferences regarding a GP’s gender and age.

However, it was not always the case that the GP had known the resident for a long time prior to them becoming a sheltered housing resident. Knowledge of the patient’s medical history was therefore not necessarily obtained. Regardless of duration of the contact between the resident and the GP, problems often arose if the resident needed a consultation and was asked to attend an appointment at the GP’s office. The transportation could be exhausting for residents with severe health conditions. A sheltered housing manager said:

It’s usually like that with this group… they are perhaps 20 years older somatically than they actually are. So, at 60, she is now … probably like an 80+. They need a lot, and there are many different issues. And I can see that going to the GP’s office is like a trip to America for them. (Participant 11)

Making home visits is a statutory duty that all GPs comply with. Because GPs have busy days and patient appointments throughout the day, home visits were often added to the afternoon after office hours. It could be an extra challenge to facilitate these if there was a long distance between the GP's office and the sheltered housing, and the GP's working day became significantly longer.

Collaboration between the GP and the sheltered housing staff was considered important for following up residents’ medical problems. However, if the GP-patient relation was newly established, GPs would have little knowledge about the patients. This was described as challenging for efficient service delivery. Study participants described occasions when GPs were requested by sheltered housing staff to provide immediate medical supervision for a resident. It could be difficult to assess the inquiry, and the GP had to spend time following it up by contacting the sheltered housing and requesting more detailed information. It was especially challenging to interpret the information when the GP did not know the staff working in the sheltered housing or their professional assessment competence.

### Model 2. The single GP model: one common/shared GP for all residents

The second model we present is what we may call the ‘single GP model’. Sheltered housing staff or management recruit one specific GP who serves all residents. There were two examples of this model in the study. This way of organizing medical services was explained by the study participants as having emerged because sheltered housing managers and staff had found it difficult to organize collaboration with many different GPs for their residents.

Study participants from the sheltered housing working within such a model argued that most residents were unable to make an individual choice of GP and too sick to contact the GP themselves. Following this, they argued that residents of old age and with complex care conditions, including dementia, were unable to manage the autonomy inherent in the GP scheme. They explained that most residents were unable to communicate about their health by themselves and needed help from staff to both book a consultation and explain their health problems to the GP. When new residents moved into the sheltered housing, they were strongly encouraged to change GP to the one that the sheltered housing collaborated with. Study participants emphasized that they would not *demand* that anyone changed GP, but they would describe the benefits residents would experience if they changed.

Furthermore, study participants working with persons with intellectual disabilities reported that they did not have language to express health concerns, and therefore staff were essential for interpreting behavioral changes which, in turn, were reported to the GP. Many residents also needed help from staff after a medical consultation to follow up what was said at the GP’s office:

Many residents need us afterwards … to explain or summarize what was said in the doctor’s office. We are almost talking about translating what has been said, so it becomes understandable… and many residents do not remember. (Participant 4)

In total, staff argued that the benefits of this way of organizing medical services for residents were greater than the disadvantage of residents not choosing their own GP.

Previous challenges with collaboration between sheltered housing staff and GPs were one motivation for establishing close collaboration with one dedicated GP. In this way, they had the opportunity to form a good collaborative relationship regarding residents, study participants said. Staff had previously spent considerable time on coordination with individual GPs, striving to learn how a particular GP thinks, plans and works. After the new model was implemented, the GP came one fixed day every week or every other week for consultations in the sheltered housing. The staff planned and prioritized residents who needed medical attention. On the day of the visit, the GP first examined these residents. Afterwards, the GP would see other residents who needed minor clarifications. The GP also often took time to talk to employees and discuss general issues with them. Over time, the employees and the GP knew each other well and learnt what to expect from each other, trusting each other’s assessments of residents.

One GP argued that the weekly visit to the sheltered housing formed a better relationship with the residents. She found that, by just greeting, observing, and meeting the residents on the way in or out of the sheltered housing. She said:

I see the patients when I am there; even if I am not supposed to see them as patients, I observe them. They know when I come, and they are sitting there… they recognize me, and they wave to me. They do not always remember that I am their doctor, but I am a familiar face, or maybe it is my voice, my movements, someone they have seen before and who they – yes – relate to. (Participant 12)

Such unplanned, frequent contact would be useful to the GP for later planned consultations.

Because the GP and staff were familiar with each other’s competence and assessments of residents’ medical needs, they could also sort out many issues over the telephone. This would decrease the need for the GP to conduct home visits and for residents to be sent to emergency wards, study participants explained.

On the other side, this model goes against the individual’s right to choose a GP for themselves. Some participants argued that ignoring this fact was a violation of basic patient rights, especially if the residents had capacity to consent. Another difficulty with this model concerns the fact that GPs seldom have the capacity to take several new patients on their lists. Those GPs who were responsible for all residents at a sheltered house in our study had taken this responsibility when they started working in the municipalities and had only a few patients on their lists.

Furthermore, an argument that came up against having one common GP for many residents was the hardship of handling the health needs of a demanding group of patients who often had complex challenges. A nurse in a sheltered house for residents with addition and psychiatric problems stated:

I think it is good that the GPs have a somewhat even distribution of this user group because it is a very… there is a lot of work with them… it is demanding, and it requires you to have the energy to handle it… So, if one GP had a long list with many patients with serious addiction and psychiatric problems, it is heavy… (Participant 3)

### Model 3. The hybrid model: a few dedicated GPs follow up the residents

The third model explained in the study can be labelled a ‘hybrid model’, that is a combination of models 1 and 2. Sheltered housing staff recruited a few GPs with whom they already had good ongoing communication and collaboration and who were known to have specific competence regarding the resident group. As in model 2, residents were encouraged to change their GPs to those recommended by the sheltered housing staff.

We found three examples of such ways of organizing medical services, all of which came into being in a less planned way than models 1 and 2. They arose because of the acknowledgement that residents needed better medical follow-up. Two emerged because sheltered housing staff and GPs found it stressful to keep track of, collaborate with, and provide help to residents who needed considerable follow-up, study participants explained. GPs that formed this informal and close collaboration with the sheltered housing were either located close by or had a special interest in the resident group, for example residents with intellectual disabilities or drug and substance abuse. A GP taking on the responsibility for residents with intellectual disabilities argued that, because he knew this group well, he was aware of which of their special medical conditions to check for – which might not necessarily be known to all GPs.

… If you have Downs syndrome, there are certain things you need to take care of, so they do not become a health problem… these are things that the sheltered housing employee does not necessarily think of as a problem. For example, vision. There is such a thing as keratoconus…. So, I look for I am systematic …and know what to do at my annual check-up. (Participant 17)

Another example of such ad hoc ‘hybrid’ organizing was found in a municipality where the municipal chief physician had initiated collaboration between the staff in a sheltered house and a few GPs. The GP and the sheltered housing staff worked closely together and had developed routines and practices for collaboration, leading to a high level of trust. A nurse in the sheltered housing said:

I think she [the GP] likes working with us… We know each other well. And she knows how we work and think. She knows what kind of observations we make and what we do before we make contact… So, when I say, ‘this resident needs a cure for a urinary tract infection,’… she knows that’s what the resident needs. (Participant 9)

The interviews suggest that sheltered housing that has formalized collaboration with a few GPs has more contact – both formal and informal – than those collaborating with a multitude of GPs. Study participants argued that such organizing meant that the GPs have comprehensive knowledge of residents on their lists. The GPs were also familiar with the sheltered housing staff and their assessments, which meant they would trust information and react to requests more easily than if they did not know them. Likewise, the sheltered housing staff knew about the GP's tight work schedule and would only ask for help from the GP if really necessary. Then the GP would know that it was a thought-through, reasoned request and reach out as soon as possible.

One challenge with this ad hoc organization and collaboration was that the GPs had busy days and were not always available and ready to reply to questions and concerns that sheltered housing staff had for residents. The request was added on top of their tasks and they ‘found time’ to accommodate it.

Table 4 summarizes the findings of our study, with an emphasis on experiences/consequences for residents, sheltered housing staff and GPs, as well as for the collaboration between providers.

**Table 4.** Study participants’ positive and negative experiences with organizing medical services from the GP in the municipalities.

		Experiences and consequences		
Model	Organizing principle	For residents	For staff in sheltered housing	For GPs
1	Each resident has their own GP	Varied. Positive if there is long-term contact and continuity but negative if there is no contact between GP and resident prior to the sheltered housing. Difficult for frail residents to access the GP’s office.	Many GPs to deal with, and they often have different work methods and approaches to residents and what steps to be taken. Difficult to get good routines for cooperation when the communication is rare.	GPs do not always know the residents. Many residents are too frail/sick to attend consultations in the GP’s office, and the GPs must make home visits. Difficult to assess the urgency and need expressed in messages about residents’ changing health conditions when GPs do not know the resident or the staff’s assessment competence.
2	One shared GP for all residents	Continuity of care to be ensured after the change of GP. Residents see the GP on a regular basis (weekly or every other week). Predictable, but residents have to be willing to choose a GP that might be new to them.	Easy to relate to, good cooperation, know each other and each other’s assessments, supervision on fixed agreed days. Predictable and can plan for communication on a set day in the week	Overview, continuity with the residents, frequent follow-up provides updated patient knowledge, receive reliable help with observations from medical professionals at the residence.
3	A few dedicated GPs follow up the residents	Confidence, the GP knows the residents well, and has good expertise about the group.	Know the doctors, get used to working together, easy to contact, have fixed appointments.	Know the residents and the homes’ employees and their professional assessment competence.

## Discussion

This paper reports from a study on how medical services provided by GPs are organized for sheltered housing residents. We have analyzed the experiences of sheltered housing staff and GPs serving residents and presented these in three main models, addressing the advantages and disadvantages of each.

From the models presented in the results section and summarized in Table 4, three topics emerge as central for further discussion: (a) the value of continuity of care, (b) residents’ right to choose their own GP, and (c) collaboration between GPs and sheltered housing staff.

### Continuity of care

In this study, we have seen that continuity of care was considered a strong argument for patients keeping their GP when moving into sheltered housing. This argument is at the core of the intention of the GP scheme. The value of knowing the patient and their history well, by having a permanent physician over time, is seen as an ideal and connected/related to positive health outcomes such as service quality and patient security/safety [8,9,11,29]. Good patient knowledge and long-term contact were seen among study participants as beneficial when patients entered old age and multimorbidity. However, our study indicated that far from all residents had long-standing contact with a GP. Evaluations have shown that the GP scheme is under pressure. Over recent years, GPs have experienced a significant increase in workload, and many now find their work situation unmanageable and are seeking other positions [19]. A shortage of GPs is detected, especially in smaller rural municipalities, and the use of substitutes and short-term contracts is widespread [30]. Statistics from the GP register confirm that the number of legal bases (hjemler) and the use of temporary workers are on the rise, largely reflecting challenges resulting from GPs having left the municipalities [30]. High GP turnover will jeopardize the ideal of continuous relations between physicians and patients/families over time.

Another factor that challenges continuity of care is the patients (themselves). As they age, they can have difficulties complying with expectations in the GP scheme: they are unable to contact the GP to book an appointment by themselves and, due to mobility problems or poor somatic or mental health, they might not be able to access the GP’s offices. In a similar manner, former studies have shown that sheltered housing residents overall have a large health burden and are unable to access health services by themselves [7]. For this reason, GPs are often requested to make home visits, which again are hard to accommodate due to an already heavy workload.

### Residents’ right to choose

A patient’s right to choose their GP is described in Patient and User Rights Act from 1999 and the individual’s right to make decisions for oneself is seen as a cornerstone in Norway. While all study participants – independent of which organizing model they had – acknowledged that sheltered housing residents choose their GP, some claimed this right to choose is of greater importance to some patients than others. For example, for sheltered housing residents with substance abuse and/or psychiatric diagnoses, the right to choose was seen as of high importance. This group of residents often had personal experiences and clear preferences regarding whom they would choose as their GP, and they might change their GP frequently.

However, old-age residents, especially those with complicated health problems, were often seen as less able to make a choice about a GP late in life. Old-age sheltered housing residents were described as very frail, and many also had cognitive challenges hampering their ability to choose for themselves. They did not have knowledge of the various GPs serving their community/municipality and needed help from staff or family members to choose. Frail or cognitively reduced residents also required help in all steps of consulting a GP, from contacting the GP and booking an appointment, to getting to the GP’s office and finally obtaining help in explaining the reason for consulting the GP and following up on messages, medication and complying with treatment.

Studies have also shown that some groups of residents/home dwelling persons might benefit from being followed up by GPs who have specific competence in certain areas [31]. A few GPs in our study stated that some groups of patients (for example, those with intellectual disabilities) were relatively small in the general population and being a GP for these residents could require special competence. Not every GP possessed this competence, and the GP with broad competence could more easily overlook symptoms or conditions typical to particular groups of residents.

### Collaboration

An ideal in contemporary healthcare delivery is integrated care, where transitions between different parts of healthcare are seamless and smooth. Coherent and coordinated care pathways are national health policy goals [32], and to achieve this goal, collaboration across and between professionals and sectors is necessary. For patients in sheltered housing, collaboration – and integration of care – on a clinical level [16] between GPs and sheltered housing staff is specifically important.

It is not surprising to find that models ii and iii – where a few GPs cooperate with sheltered housing staff – had developed and valued a high level of close collaboration. In these models, GPs and sheltered housing staff knew each other well (and nurtured their cooperation regularly). Numerous studies have documented the value of shared knowledge among health personnel and mutual respect for each other’s competence as a central factor in collaboration [33]. Model ii seems to have developed specifically out of a need to resolve difficulties in collaboration. Over time, GPs and sheltered housing staff developed knowledge of one another’s competence, regularly discussing work procedures and trusting each other. This strengthened clinical care integration [16].

As seen in the results section, in model ii, residents are regularly supervised by a GP who follows them up regularly. This seems especially important for residents with extensive healthcare needs. The sheltered housing staff felt it was time-saving and good to be able to work with one GP. A member of staff in one sheltered house claimed that having a common GP who knew the resident group well had prevented hospitalizations for some residents. This was due to the GP acquiring good patient knowledge and good help from employees to make assessments. Studies indicate that investing in close collaborations and integrated care might increase cost-effectiveness and patient experience, but the evidence is uncertain [17,18]. Although our study was not designed to answer such questions, our findings point to increased satisfaction among providers. In current times, when workforce shortages are becoming more frequent, one should take into account providers’ experiences, in addition to more traditional measures like patient outcomes and experiences.

## Conclusion

The study has shown that residents in round-the-clock staffed sheltered housing constitute a varied group that generally has substantial medical assistance needs. Models ii and iii seem to provide better integrated services, with more and better interaction between medical professionals and residents. Given the user group’s lack of autonomy to manage their own medical care needs and make decisions, models with fewer GPs seem to provide a clearly better medical professional offer. The GP system is built on the premise that patients are able to initiate contact when needing assistance; when residents can no longer do this, someone from the sheltered housing needs to takes responsibility for the contact and following up on communication and treatment. However, moving towards such an organising of the medical services for sheltered housing residents could have implications for GPs’ workload and competence needs. Future work for improving services for sheltered housing residents should include systematic testing of different models and their effects on both residents, providers and on social economic measures.

### Limitations

This is a limited qualitative study that aimed to explore a field where there is little knowledge. To explore this topic further, more detailed studies are needed. The study was originally planned with a design where we would interview both sheltered housing employees and GPs in the same area. Due to the COVID-19 pandemic, it was difficult to recruit GPs, and we therefore also included GPs outside the municipalities where the sheltered housing units were located. This approach prevented us from obtaining the experiences of both GPs and staff regarding the medical follow-up in the same sheltered housing, but it has given us insight into experiences from more municipalities and sheltered housing than planned.

A weakness with the study is that the largest municipalities in Norway were not included. We may therefore have missed other ways of organizing and experiences with the medical services in even more complex organizations than the municipalities that were included in the study. Future studies should make sure to include municipalities of all sizes. Furthermore, to gain a broader understanding of the medical services for sheltered housing residents, studies should be designed to include the experiences of residents and their next of kin.
